# Evaluating the Impact of IMU Sensor Location and Walking Task on Accuracy of Gait Event Detection Algorithms

**DOI:** 10.3390/s21123989

**Published:** 2021-06-09

**Authors:** Wesley Niswander, Kimberly Kontson

**Affiliations:** US Food and Drug Administration, Center for Devices and Radiological Health, Office of Science and Engineering Labs, Silver Spring, MD 20993, USA; wniswand@terpmail.umd.edu

**Keywords:** gait, wearables, inertial measurement units

## Abstract

There are several algorithms that use the 3D acceleration and/or rotational velocity vectors from IMU sensors to identify gait events (i.e., toe-off and heel-strike). However, a clear understanding of how sensor location and the type of walking task effect the accuracy of gait event detection algorithms is lacking. To address this knowledge gap, seven participants were recruited (4M/3F; 26.0 ± 4.0 y/o) to complete a straight walking task and obstacle navigation task while data were collected from IMUs placed on the foot and shin. Five different commonly used algorithms to identify the toe-off and heel-strike gait events were applied to each sensor location on a given participant. Gait metrics were calculated for each sensor/algorithm combination using IMUs and a reference pressure sensing walkway. Results show algorithms using medial-lateral rotational velocity and anterior-posterior acceleration are fairly robust against different sensor locations and walking tasks. Certain algorithms applied to heel and lower lateral shank sensor locations will result in degraded algorithm performance when calculating gait metrics for curved walking compared to straight overground walking. Understanding how certain types of algorithms perform for given sensor locations and tasks can inform robust clinical protocol development using wearable technology to characterize gait in both laboratory and real-world settings.

## 1. Introduction

Evaluation of human gait is important for the diagnosis of gait pathologies caused by certain medical conditions as well as the rehabilitation of those gait pathologies [1]. The use of wearable sensors, such as inertial measurement units (IMUs), to detect gait events and calculate spatiotemporal gait metrics in both healthy populations as well as those with gait pathologies is an emerging area of research and clinical relevance [2]. There are several studies that have focused on the use of IMUs to characterize gait metrics in clinical populations such as individuals with Parkinson’s disease, stroke, and spinal cord injury [2,3,4]. The most commonly reported gait events are heel strike (initial contact) and toe off (final contact) which enable the calculation of spatiotemporal metrics including cadence, stride time, stance time, and swing time [3,5,6,7,8].

Despite the interest in using IMUs to measure spatiotemporal gait metrics, there is little consensus on the best algorithmic approach to accomplish this. One research group recently published a literature review that identified 17 different algorithms for identifying heel strike and toe off gait events during over ground walking [2] and conducted an analysis of these 17 gait event detection algorithms during gait in water [9]. Many of these algorithms use maxima/minima of the acceleration and/or rotational velocity signals in one plane [3,4,5,7,10,11,12] or determine the zero crossing of a relevant signal to identify the heel strike and toe off gait events [8,13]. Adding further convolution, many algorithms were developed to work with sensors mounted to specific locations on the lumbar spine, shank, and foot. Although limited previous works have noted that sensors mounted on the lumbar spine generally perform worse than sensors mounted on the shank or foot for gait event detection applications [2], a robust systematic evaluation of the impact of different sensor locations on the accuracy of various gait event detection algorithms is poorly documented [14] with very little information about differences between shank and foot mounted sensors.

Additionally, most studies on IMU algorithm development or assessment for gait focus on straight over ground or treadmill walking [5,7,10,11,15,16], with a few evaluating specific algorithms during turning [4,13]. Daily ambulation in a real-world context requires a wider range of gait activities than straight level walking, making the investigation of different walking motions critical in gait metric measurement. Individuals are faced with obstacles in the form of curbs, stairs, cones, or other people, which forces them to deviate from the typical straight-line walking recorded during laboratory testing. Studies have shown that more than 30% of walking time during community ambulation is spent along curved trajectories [17,18]. However, it is less clear how these curved trajectories impact the accuracy of various gait detection algorithms. One recent study investigated the impact of curved walking, turning, and cognitive load on detection performance of gait events using shank-mounted IMUs [19]. Results showed that detection performance was adequate for curved walking, but there were more false events detected during turning. While this study begins to address the paucity of research on this topic, it was limited to a single sensor location and algorithm using angular velocity.

Given these gaps in the current literature, the objective of this study was to systematically investigate the performance of a cross-section of previously used algorithms at different IMU sensor mounting locations on the foot and shank to determine the relationship between algorithm and location. Three-dimensional acceleration and rotational velocity data were collected from individuals performing two distinct walking tasks in order to determine the impact of these walking tasks on algorithm performance. The results from this study will bolster the body of knowledge on IMU gait measurement and identify algorithm and location combinations with higher accuracy. Furthermore, the addition of a curved walking task makes the results of this study more applicable to real-world ambulation. This has implications in both research and clinical settings. These results will give researchers insight into the most appropriate algorithm/sensor location combination when capturing data outside of laboratory environments, which will allow for the monitoring of more activities observed in laboratory settings [20]. In terms of clinical implementation, these results could facilitate the adoption of at-home monitoring of patients. At-home monitoring using IMUs is gaining interest, making the demonstration of gait event detection accuracy with IMUs during a wide range of gait motions a topic of importance [21,22].

## 2. Materials and Methods

### 2.1. Participants

A convenience sample of seven participants were recruited (4M/3F; 26.0 ± 4.0 years of age). All participants provided written informed consent prior to participation. The study was conducted in accordance with the Declaration of Helsinki, and the protocol was approved by the U.S. FDA Institutional Review Board (# 2019-CDRH-002). Participants were asked to wear flat, close-toed shoes. For participants who did not have proper footwear, two-strap Velcro sandals were provided.

### 2.2. Data Acquisition

**Figure 1:**
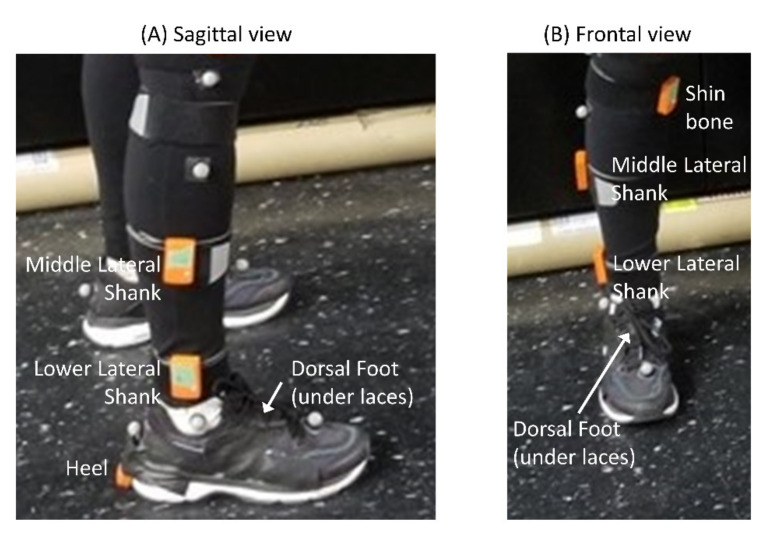
Depiction of sensor location on a participant from the (**A**) sagittal view and (**B**) frontal view. The names of each sensor are shown next to the sensor.

The Xsens MTw Awinda IMU system was used to capture 3-axes accelerometer and rotational velocity data from participants as they performed two walking tasks. Data from these sensors were processed using the accompanying MT Manager software. The human 46.1 filter was used and Fs = 60 Hz. Based on results from a literature search, five common sensor locations on the foot and shank were identified: shin bone (shin), middle lateral shank (MLS), lower lateral shank (LLS), heel, and dorsal foot (DFoot) (Table 1). Each participant was prepared for the experiment by securely placing sensors at each location on the right leg (see Figure 1). Although other locations on the upper thigh and lower torso were identified as common locations for IMU sensors in previous work [23], Panebianco et al. found that torso-based algorithms generally performed worse than shank- or foot-based algorithms [2], so only shank and foot locations were selected for investigation in this study. Additionally, very few studies report on thigh-based algorithms.

A 16-foot instrumented pressure walkway from Protokinetics was also used in the study as a reference system for determining heel strike (HS) and toe-off (TO) gait events. Data on sensors activated by the right and left feet during gait were exported and used to determine HS and TO gait events.

### 2.3. Walking Tasks

Participants were asked to perform two walking tasks: straight over ground walking and obstacle navigation. The straight over ground walking task required participants to walk in a straight line at a self-selected pace from one end of the 16′ mat to the other. Once participants reached the end of the mat, they turned around off the mat and continued walking. Each participant walked across the mat six times. For the obstacle navigation task, participants were again asked to walk from one end of the mat to the other six times, but three standard sized shoe boxes were placed as obstacles at equal intervals on alternating edges of the mat. The participant was asked to avoid these obstacles, which resulted in a serpentine walking path. This was done to investigate the effects of non-straight walking on the accuracy of the algorithms.

### 2.4. Gait Event Detection Algorithms

The same literature search to determine common sensor locations on the lower body was used to identify common algorithms used to detect gait events using IMUs. The literature reporting on the use of accelerometer or rotational velocity data to detect HS and TO gait events was considered. This literature search was not meant to be exhaustive, but instead provide a cross section of commonly used algorithms.

As a result of this literature search, there were five main algorithms investigated in this study. The implementation of algorithms was performed in MATLAB (MathWorks). A brief overview of each type of algorithm is provided here. For additional details, we encourage the reader to review the referenced manuscripts.

The first type of algorithm looks at rotational velocity minima. Multiple sources reported using variations of these algorithms [4,5,10,11,33,38]. These algorithms look at minima in the medial-lateral rotational velocity signal. These minima occur before and after a large peak occurring during mid-swing and are used to estimate the timing of HS and TO.

The second type of algorithm looks at rotational velocity zero crossing. Again, the medial-lateral axis of the signal is used. This algorithm looks for where the rotational velocity signal crosses zero before and after the mid-swing peak. A few sources have utilized this approach in determining HS and TO [7,8,13,39].

The last three types of algorithms used acceleration features to identify gait events. Some references used superior-inferior and anterior-posterior acceleration peaks to identify HS and TO [7,40], while others used a combination of acceleration and rotational velocity data [3,8]. A brief description of these algorithms applied to the current study follows, along with explanations for any deviations in the algorithms from the reported literature.

The third type of algorithm is referred to as the SIacc/APacc (superior-inferior acceleration/anterior-posterior acceleration) method. This method is based on the Jasiewicz method which requires an estimate of drift corrected angle to find the times of maximum plantar/dorsiflexion [7]. This was accomplished in Jasiewicz et al. 2006 using a zero-velocity update technique (ZUPT) which has been described by multiple sources [7]. This method is most applicable to measurement on the foot but inadequate when estimating angles for the shank because the shank never stops moving during stance. As such, a modified ZUPT method was used when implementing SIacc/APacc for the two sensors on the shank. This modified method estimates points of flat foot as the halfway point between subsequent mid-swing peaks in the medial-lateral signal. Once the angle estimate is found, TO is obtained as a minimum in anterior-posterior acceleration near peak plantar flexion, and HS is found as a minimum in superior-inferior acceleration near peak dorsiflexion.

The fourth type of algorithm is referred to as the MLvel/APacc (medial-lateral rotational velocity/anterior-posterior acceleration) method. This method, based on Trojaniello 2014, detects HS as a rotational velocity minimum and TO as an anterior-posterior acceleration minimum [3]. For added robustness, regions of trusted stance and trusted swing are defined where gait events cannot be detected [3]. However, the definition of these regions required data from sensors on both legs. The current study only captured data on the right leg, so only a trusted swing can be defined here. Trusted swing is defined as times when the medial-lateral rotational velocity of the sensor is greater than 20% of its maximum value at mid-swing. HS and TO are then found on either side of the current mid-swing peak at times when the rotational velocity is less than this 20% threshold.

The fifth type of algorithm is referred to as the APacc/MLvel (anterior-posterior acceleration/ medial-lateral rotational velocity) method, based on Rampp 2014 [8]. The TO point is found as a ML rotational velocity zero crossing. The HS point is found as a maximum anterior-posterior acceleration within a search window 50ms before and 20ms after a medial-lateral rotational velocity minimum. This method varies from Rampp 2014 in which strides are segmented using an algorithm based on subsequent dynamic time warping [8]. Here, gait event timing is determined solely on the proximity and location of signal features relative to mid-swing peaks.

### 2.5. Sensor Calibration

A sensor-to-body calibration was used to transform IMU data into data in terms of the anterior-posterior (AP), medial-lateral (ML), and superior-inferior (SI) anatomical directions. The calibration approach used here has been previously described [23,41]. Briefly, this approach utilizes two static poses to determine the direction of anatomical axes using gravitational acceleration. In the first pose, participants stand still in a neutral posture with their toes pointed forward. The gravity vector obtained during this period is normalized and assumed coincident with the SI direction. The participant then adopts a seated pose in which they lean back and extend their legs forward with their heels touching the ground. Care is taken that the principal axes of all body segments lie parallel to the sagittal plane. The gravity vector obtained during this pose is normalized and assumed to lie in the sagittal plane. The ML vector is estimated by a cross product of this vector and the SI vector. A further cross product between the SI and ML vectors yield the AP vector and another cross product between the AP and SI vectors redefine the ML vector to ensure mutual orthogonality. These three-unit vectors are used to construct direction cosine matrices and are used to transform the accelerometer and gyroscope data using equation 1. Here [*x*,*y*,*z*] is a vector (either acceleration or rotational velocity), and [*x’*,*y’*,*z’*] is the same vector in terms of the anatomical coordinate system. Vectors *a*, *b*, and *c* are the SI, ML, and AP unit vectors, respectively.

(1) $$\left[\begin{array}{c} x^{\prime }\\ y^{\prime }\\ z^{\prime } \end{array}\right]=\left[\begin{array}{ccc} a_{1} & b_{1} & c_{1}\\ a_{2} & b_{2} & c_{2}\\ a_{3} & b_{3} & c_{3} \end{array}\right]\left[\begin{array}{c} x\\ y\\ z \end{array}\right]$$

### 2.6. Data Analysis

The accuracy of the algorithms at detecting gait events was compared against a pressure sensing walkway. A 16′ walkway from Protokinetics was used (Protokinetics LLC, Havertown, PA, USA). Instrumented walkways have been used extensively in gait studies and previously validated against 3D optical motion capture systems [3,15,39,42].

The relative accuracy of the different algorithms at detecting gait events was compared against the instrumented walkway. This was done for all five algorithms at all five sensor locations. Three gait metrics were calculated: stance time, swing time, and stride time. Stride time is defined as the time between subsequent heel strikes. The stance time and swing time metrics were calculated for steps defined between heel strikes (i.e., heel strike, toe off, heel strike). Stance time is given as the time between HS and TO, and swing time is given as the time between TO and HS. The first right foot HS from the instrumented walkway was visually aligned with the first HS as measured on the DFoot IMU sensor using ML rotational velocity. Since all IMU sensors collected data synchronously, this alignment applied to all IMU sensors. In some cases, the participant may have walked off the edge of the mat, leaving only a partial footfall captured by the instrumented walkway. There may also be some confounding effects from starting and stopping at the edges of the instrumented walkway. Therefore, to eliminate confounding effects from partial footfalls and starting/stopping, the first and last gait events detected on the mat were removed when calculating the gait metrics; all remaining gait events were captured by both the IMU and instrumented walkway and included in the analysis. Instead of comparing the identified frame for a HS or TO event from the IMUs using a given algorithm to the identified frame for a HS or TO event from the instrumented walkway, the gait metrics for each modality were calculated separately. That is, stance time, swing time, and stride time were calculated on a step-by-step basis for each IMU sensor/algorithm combination and for the instrumented walkway.

The error between the gait metrics derived from the IMUs and instrumented walkway reference system was quantified using root mean square error (RMSE) and mean absolute bias. The derived metrics across all subjects were taken into account for these calculations. Mean absolute bias was calculated as an average of the absolute difference between the IMU-derived gait metric and instrumented walkway-derived gait metric. Similarly, the RMSEs were calculated as the square root of the average of squared difference between IMU-derived gait metric and the instrumented walkway-derived gait metric. These error calculations were done for each task, algorithm type, and sensor.

## 3. Results

Mean absolute bias and RMSE values are presented in tabular format (Table 2, Table 3 and Table 4) for both straight over ground walking and obstacle navigation tasks. The mean absolute bias values and RMSE values are reported in units of milliseconds (ms) to facilitate comparisons with previous studies.

For stance time during straight over ground walking, the lowest RMSE and mean absolute bias values were generated using the MLvel/APacc algorithm (type 4) and the middle lateral shank sensor (RMSE: 22.2 ms; Abs Bias: 17.9 ± 13.2 ms) (Table 2). In general, however, the dorsal foot sensor tended to generate lower RMSE and mean absolute bias values independent of the algorithm. The heel sensor location generated substantially higher RMSE and absolute bias values for the SIacc/APacc (type 3) and MLvel/APacc (type 4) algorithms. Similar patterns were seen for the obstacle navigation task, where the middle lateral shank generated the lowest RMSE and mean absolute bias values using the MLvel/APacc algorithm (type 4) (RMSE: 28.4 ms; Abs Bias: 22.1 ± 17.8). The dorsal foot sensor location also appeared to be the most robust location for all algorithm types. In comparing the accuracy across the two walking tasks, the SIacc/APacc (type 3) and MLvel/APacc (type 4) algorithms generated much higher RMSE and mean absolute bias values during the obstacle navigation task for the heel and lower lateral shank sensor locations. Specifically, RMSEs were 23.8 ms and 51 ms higher for the heel and lower lateral shank locations, respectively, for the obstacle navigation task MLvel/APacc (type 4) algorithm. Mean absolute biases were 27.5 ms and 24 ms higher for the heel and lower lateral shank locations, respectively, for the obstacle navigation task MLvel/APacc (type 4) algorithm. For the SIacc/APacc (type 3) algorithm applied to the lower lateral shank, RMSE and mean absolute bias values were 29.2 ms and 19.1 ms higher during the obstacle navigation task.

The results for swing time show the same pattern as the result for stance time, with the middle lateral shank sensor and MLvel/APacc algorithm (type 4) yielding the lowest RMSE and mean absolute bias values across tasks (Table 3). The dorsal foot sensor location was generally associated with lower RMSE and mean absolute bias values independent of algorithm. Similar patterns were seen when comparing the two walking tasks, with the SIacc/APacc (type 3) and MLvel/APacc (type 4) algorithms generating much higher RMSE and mean absolute bias values during the obstacle navigation task for the heel and lower lateral shank sensor locations. Specifically, RMSEs were 24.8 ms and 44.2 ms higher for the heel and lower lateral shank locations, respectively, for the obstacle navigation task MLvel/APacc (type 4) algorithm. Mean absolute biases were 27.2 ms and 19.7 ms higher for the heel and lower lateral shank locations, respectively, for the obstacle navigation task MLvel/APacc (type 4) algorithm. For the SIacc/APacc (type 3) algorithm applied to the lower lateral shank, RMSE and mean absolute bias values were 30.2 ms and 22.1 ms higher during the obstacle navigation task.

Table 4 shows the RMSE and mean absolute bias results for stride time. For this metric, the heel sensor yielded the lowest RMSE and mean absolute bias results using the velocity minima algorithm (type 1) for both the straight over ground walking task (RMSE: 9.2 ms; Abs bias: 7.2 ± 5.6 ms) and the obstacle navigation task (RMSE: 9.4 ms; Abs bias: 5.6 ± 7.5 ms). In general, the heel sensor location was the most robust against the different types of algorithms for both walking tasks. The lower lateral shank sensor location with the APacc/MLvel algorithm (type 5) generated the highest RMSE and mean absolute bias values for both the straight over ground walking (RMSE: 70.4 ms; Abs bias: 51.9 ± 47.5 ms) and obstacle navigation task (RMSE: 77.2 ms; Abs bias: 58.5 ± 50.3 ms).

## 4. Discussion

The use of wearable sensors such as IMUs to quantify gait characteristics in both healthy and clinical populations is increasing. As such, algorithms to interpret these wearable data are emerging. Most algorithms use the 3D acceleration and/or rotational velocity vectors from IMU sensors to identify gait events (i.e., toe-off and heel-strike). These gait events are then used to derive temporal gait metrics that can inform clinical diagnoses or assessment. However, a clear understanding of how sensor location on the body and the type of walking task being performed effect the accuracy of gait event detection algorithms is lacking. The overall goal of this study was to conduct a robust, systematic evaluation of the impact of sensor location and walking task on the accuracy of five common gait event detection algorithms. A discussion of the results as well as clinical relevance follows.

For all clinical measurement tools, there are many potential sources of error. These errors can result from variations between and within patients, variations in how an observer sets up a measurement device, and error in the measurement device itself [43,44]. The current study addresses knowledge gaps in the latter source of measurement error. Minimal measurement error is important for all measurement tools, especially given the potential of wearable systems to track disease progression or diagnose a disease or condition. It is important to be able to identify and quantify error associated with clinical measurement tools. In the context of this paper, the magnitude of error introduced by different algorithms and different sensor locations has important implications in the implementation of wearables for clinical purposes as well as the interpretation of the results. The RMSE and bias values presented in this study can inform sensor location and algorithm type combinations for a given gait metric. It is generally well understood that any measured change within a patient or difference between two populations needs to be larger than the error associated with a measurement to have confidence in the clinical measurement [45]. By understanding the error compared to a gold standard for these different combinations and with general knowledge about the expected difference between populations (e.g., healthy vs disease) for a given metric, an appropriate sensor location/algorithm combination can be selected. For example, a recent study found stance time between younger healthy adults and individuals with Parkinson’s disease to be 0.63 ± 0.001 s and 0.71 ± 0.03 s, respectively, when using an instrumented walkway reference system [46]. If IMU wearable systems are used in these same clinical populations, the error of the measurement device should be smaller than the expected difference between populations to have confidence in the clinical comparison of these groups.

While several representative algorithms were tested, it was not possible to include every possible algorithm. Instead, this paper focused on generalizing commonly seen algorithms used on foot and shank IMU sensor data to augment a recent study by Panebianco et al. in which a systematic review of publicly available literature compared 17 different algorithms for sensors mounted at the lumbar spine, lower lateral shank, and dorsal foot locations [2]. The current study sought to expand upon this topic by examining additional sensor mounting locations on the heel and shank and by including a curved walking task that elicits motion more commonly found in real world ambulation. Due to findings from previous work indicating lumbar spine-based algorithms to be less accurate than their shank and foot counterparts [2], lumbar spine algorithms were ignored. These algorithms are meant to serve as a cross section of algorithms examining zero crossing and peak identification features using both rotational velocity and acceleration signals.

The RMSE and mean absolute bias values reported in this study are similar to those previously reported using pressure sensing walkways, force plates, and optical motion capture as reference systems, yielding confidence in our experimental protocol. Another study compared an algorithm for detecting gait events using a sensor mounted on the anterior shank against force plate and optical motion capture data. In this case, data were processed using the Hreljac–Marshall algorithm, which determines TO and HS events relative to the large positive peak in the medial-lateral rotational velocity signal during midswing [5]. Stride, stance, and swing time non-mean absolute biases for straight over ground walking (average speed 1.10 m/s) reported in this study were −6.35ms, 35.43ms and −58.04ms, respectively [5]. The rotational velocity minima algorithm (type 1) used in this study was most similar to this algorithm. Results from Table 2, Table 3 and Table 4 show mean absolute bias values 10.7 ± 11.5 ms, 61.0 ± 24.9 ms, and 57.6 ± 21.5 ms for stride, stance, and swing time, respectively, for this algorithm at the shin bone location during straight over ground walking. Differences exist between the stance time reported in the current and previous study that could be due to the speed at which subjects walked across the mat and the exact location of the sensor.

Trojaniello et al. found mean absolute bias values of 10, 22, and 22 ms for stride, stance, and swing times, respectively, in healthy elderly adults during straight overground walking [3]. In that study, an instrumented walkway (GaitRite) was used as a comparator system with an IMU mounted to the lower lateral shank [3]. At the lower lateral shank location, the MLvel/APacc algorithm (type 4), which was derived from the method used in the Trojaniello et al. study, produced similar results (9.4 ± 6.9 ms, 22 ± 18.7 ms, and 23.3 ± 19 ms for stride, stance, and swing times, respectively). Although this algorithm was developed for the lower lateral shank location, it is interesting to note that the MLvel/APacc algorithm (type 4) produced even lower mean absolute biases for the middle lateral shank location (8.8 ± 6.1 ms, 17.9 ± 13.2 ms, and 18.9 ± 14.3 ms, for stride, stance and swing time, respectively). This observation shows the robustness of some algorithms against sensor location. On the contrary, we also see limitations in this same algorithm when applied to a sensor location on the heel. For the MLvel/APacc algorithm (type 4), the RMSe and mean absolute bias values all nearly exceeded 200 ms for swing and stance time metrics at the heel location.

**Figure 2:**
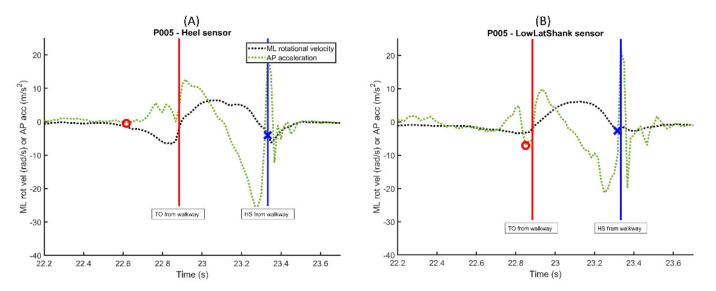
ML rotational velocity and AP acceleration plots for a single subject and a single step for the (**A**) heel sensor location and (**B**) lower lateral shank location. Red circles and blue x’s denote the identification of TO and HS by the MLrotvel/APacc algorithm (type 4), respectively.

When considering the reason for the robust performance of the MLvel/APacc algorithm across all sensors except the heel, the approach to defining regions of trusted swing where gait events could not be detected plays a role [3]. As mentioned earlier, the experimental protocol used in the current study only defines trusted swing since only the right foot was instrumented. By defining these trusted swing regions, the potential for erroneous identification of gait events decreases. The algorithm to define trusted swing (i.e., time interval where ML rotational velocity is larger than 20% local maximum value) has been shown to be consistent across populations with normal and pathological gait for sensors located on the shank and dorsum of the foot [4,11]. These defined regions of interest appear to be applicable to most sensor locations. However, applying these regions of interest definitions at the heel sensor location can result in inaccurate gait event detection. After examining the AP acceleration data for the heel and lower lateral shank sensors, it appears as if the heel sensor acceleration oscillates in a general positive direction in the instances prior to the TO gait event, which would make the location of the AP acceleration minimum in this region of interest much earlier than the TO event detected when using the other sensors. An example of a single step from one subject is shown in Figure 2, where the red circles and blue x’s denote the gait event location for TO and HS, respectively, as determined by the MLrotvel/APacc algorithm (type 4).

One unique aspect of this work is the inclusion of a curved motion walking task elicited by requiring subjects to navigate through obstacles. Many studies fail to examine human gait during tasks other than straight level walking [5,7,10,11,15,16]. Since straight over ground walking is an idealized motion, it cannot be representative of all motions which may challenge these algorithms during normal ambulation. By including a task which involves curved walking trajectories, the results from this study provide preliminary data to support the use of certain algorithms and sensor locations in order to move closer to measuring gait in “real world” environments. When comparing the RMSE and mean absolute bias values between the two walking tasks, results were fairly consistent for all sensor locations and algorithms when calculating the stride time gait metric (Table 4). However, when calculating swing/stance time, algorithm performance degraded for certain algorithm types for the heel and lower lateral shank sensors when going from straight overground walking to a curved walking path (Table 2 and Table 3).

Despite the extremely high RMSE and mean absolute bias values for the MLvel/APacc algorithm (type 4) at the heel location, this algorithm appears to be the most robust against sensor location for both straight overground walking and curved walking paths. The middle lateral shank location produced some of the lowest RMSE values for stride, stance, and swing times using this algorithm for both walking tasks, suggesting the MLvel/APacc algorithm used with a sensor mounted on the middle lateral shank location may produce the lowest error overall, especially when challenged with curved walking motions.

There are a few limitations with the current work. Many studies include the use of filtering techniques, with interest being drawn to wavelet transforms in a few studies [16,27]. This study did not examine the effects of data filtering. Panebianco et al. evaluated the accuracy and repeatability of algorithms exploiting different filters. In terms of stance time estimates, it was reported that shank mounted IMUs were more accurate using a raw signal but more repeatable using wavelet transforms [2]. For foot mounted IMUs, there was no significant difference between stance time estimates using raw or IIR filtered data [2]. There does not appear to be strong evidence that this kind of filtering will increase the accuracy of gait metrics, so the topic was not included in the current study. This study also limited the investigation of algorithms to five commonly cited algorithms in the current literature. While this research effort significantly expands on our understanding of algorithm accuracy, other types of algorithms for foot and shank-mounted sensors should be assessed using this framework. The data collected and analyzed in this study was from individuals without gait impairment. However, it is important to understand how these algorithms perform in patient populations impacted by gait. Future work will focus on applying this methodology to individuals with gait impairments.

## 5. Conclusions

The accuracy and optimization of techniques for monitoring and evaluating gait is crucial for making this technology a useful tool for clinical use. The current work looked at three factors that are expected to introduce error into gait metric measurements: algorithm used to detect gait events, the locations on body segments where the sensors are mounted, and the type of walking task. The results can be used to inform researchers and clinicians of ideal sensor placements for specific gait metrics in both laboratory and real-world settings.

## Figures and Tables

**Table 1:** Sensor locations used based on literature search and Xsens recommendations on the shank and foot.

Segment	Abbreviation	Description	Sources
Shank	Shin	*Shin Bone*: hard surface of tibial bone, below the knee and above the thickest part of the calf.	* Xsens recommendation
	MLS	*Middle Lateral Shank*: lateral shank, halfway between the knee and ankle.	Laudanski 2013 [24], Kong 2013 [21]
	LLS	*Lower Lateral Shank*: lateral shank, 5cm above the lateral malleolus.	Panebianco 2018 [2], Vargas-Valencia 2016 [25], Guo 2012 [26], Trojaniello 2014 [3], Khandelwal and Wickström 2014 [27], Storm 2016 [12]
Foot	Heel	Adhered to heel on the back of participant’s shoe	Kwakkel 2007 [28], Anwary 2018 [29], Khan 2017 [30], Lau 2008 [31], Rebula 2013 [32], Gouwanda 2016 [33]
	DFoot	*Dorsal Foot*: under the tongue of the participant’s shoe, approximately over the distal end of the third and fourth metatarsal bones.	Laudanski 2013 [24], Panebianco 2018 [2], Barrois 2016 [22], Var-gas-Valencia 2016 [25], Bourgeois 2014 [34], Guo 2012 [26], Hsu 2014 [35], Tadano 2013 [36], Kong 2013 [21], Scapellato 2005 [37], Kwakkel 2007 [28], Anwary 2018 [29], Jasiewicz 2006 [7], Ferrari 2016 [15], Mariani 2012 [13]

* Xsens recommendations can be found at https://tutorial.xsens.com/?_ga=2.262884484.968323122.1607367296-1094755908.1552499035 (accessed on 20 January 2021).

**Table 2:** Stance time RMSE and mean absolute bias values (standard deviation) for the straight over ground walking task and the obstacle navigation task reported in milliseconds. Algorithm type is represented by each row; sensor location is represented in each column. Bold text indicates an RMSE/mean absolute bias value was lowest across algorithms for a given sensor location. Italicized text indicates an RMSE/mean absolute bias value was lowest across sensor locations for a given algorithm.

		Straight over Ground Walking					
Stance Time	Algorithm Type		Dorsal Foot	Heel	Lower Lateral Shank	Middle Lateral Shank	Shin Bone
	(1) Velocity Minima	RMSE Abs Bias	90.3	74.7	58.6	*56.3*	65.9
			87.6(22.1)	71.7(20.8)	*52.5(26)*	52.8(19.3)	61(24.9)
	(2) Velocity Zero Crossing	RMSE Abs Bias	*61.8*	64.2	87.3	92.6	90.3
			*58.5(20)*	60.2(22.1)	84.9(20.3)	90.7(18.6)	88.5(17.7)
	(3) SIacc/APacc	RMSE Abs Bias	*40.3*	206.9	58.7	51.7	51.8
			***34.4(21)***	190.6(80.6)	41.5(41.5)	40.9(31.6)	45.6(24.5)
	(4) MLvel/APacc	RMSE Abs Bias	50.9	221.8	**28.9**	***22.2***	**40.6**
			45.7(22.4)	196.9(102.3)	**22(18.7)**	***17.9(13.2)***	**32.4(24.5)**
	(5) APacc/MLvel	RMSE Abs Bias	***40.2***	**47.5**	74.8	69.4	48.9
			*36.8(16.2)*	**44.9(15.5)**	62.5(41.0)	64.0(26.9)	46.4(15.5)
		**Obstacle navigation**					
	Algorithm Type		Dorsal Foot	Heel	Lower Lateral Shank	Middle Lateral Shank	Shin Bone
	(1) Velocity Minima	RMSE Abs Bias	93.2	74.8	75.8	*64.9*	73.8
			90.2(23.7)	71.8(21)	68.1(33.3)	*60.6(23.1)*	67.8(29.1)
	(2) Velocity Zero Crossing	RMSE Abs Bias	*50.5*	51.3	77.2	82.8	75.8
			*47.7(16.6)*	48.3(17.3)	74.6(19.7)	80.7(18.5)	72.2(23.2)
	(3) SIacc/APacc	RMSE Abs Bias	*48.5*	214.5	87.9	54.9	53.1
			*41.2(25.7)*	194.0(91.5)	60.6(63.6)	43.7(33.2)	41.7(32.9)
	(4) MLvel/APacc	RMSE Abs Bias	55.0	245.6	79.9	***28.4***	**39.2**
			50.0(22.8)	224.4(99.8)	**46.0(65.4)**	***22.1(17.8)***	**32.2(22.4)**
	(5) APacc/MLvel	RMSE Abs Bias	***35.7***	**44.1**	**60.3**	70.2	45.8
			***32.4(14.8)***	**41.6(14.8)**	47.6(37.1)	64.3(28.1)	42.7(16.6)

**Table 3:** Swing time RMSE and mean absolute bias values (standard deviation) for the straight over ground walking task and the obstacle navigation task reported in milliseconds. Algorithm type is represented by each row; sensor location is represented in each column. Bold text indicates an RMSE/mean absolute bias value was lowest across algorithms for a given sensor location. Italicized text indicates an RMSE/mean absolute bias value was lowest across sensor locations for a given algorithm.

		Straight over Ground Walking					
Swing Time	Algorithm Type		Dorsal Foot	Heel	Lower Lateral Shank	Middle Lateral Shank	Shin Bone
	(1) Velocity Minima	RMSE Abs Bias	93.1	72.4	60.0	*53.7*	61.4
			91.3(18.1)	69.5(20.4)	51.9(30.1)	*50.9(17.1)*	57.6(21.5)
	(2) Velocity Zero Crossing	RMSE Abs Bias	*62.0*	64.3	87.5	92.7	90.2
			*59.5(17.6)*	60.9(20.5)	85.5(18.6)	91.3(16)	88.9(15.3)
	(3) SIacc/APacc	RMSE Abs Bias	*43.5*	207.8	50.9	50.0	52.9
			36.4(23.9)	191.5(80.6)	*35.2(36.8)*	40.6(29.2)	47.8(22.7)
	(4) MLvel/APacc	RMSE Abs Bias	51.2	219.5	**30.0**	***23.7***	**36.3**
			46.3(22)	195.9(99.0)	**23.3(19.0)**	***18.9(14.3)***	**28.9(21.9)**
	(5) APacc/MLvel	RMSE Abs Bias	***37.8***	**49.5**	70.6	75.7	52.4
			***34.8(14.7)***	**47.3(14.4)**	58.3(39.7)	70.0(28.9)	50.2(14.9)
		**Obstacle navigation**					
	Algorithm Type		Dorsal Foot	Heel	Lower Lateral Shank	Middle Lateral Shank	Shin Bone
	(1) Velocity Minima	RMSE Abs Bias	90.4	75.0	**65.0**	*59.2*	65.0
			87.4(23)	72.0(21.1)	57.6(30.1)	*55.3(21.1)*	60.5(23.9)
	(2) Velocity Zero Crossing	RMSE Abs Bias	*56.5*	56.9	82.7	87.9	81.7
			*53.5(18)*	53.7(19.1)	80.5(19.1)	86.3(17)	78.8(21.7)
	(3) SIacc/APacc	RMSE Abs Bias	*43.7*	206.8	81.1	55.0	50.7
			*36.5(24.2)*	187.2(87.9)	57.3(57.4)	41.5(36.0)	41.8(28.6)
	(4) MLvel/APacc	RMSE Abs Bias	53.6	244.3	74.2	***31.0***	**40.8**
			48.2(23.4)	223.1(99.4)	**43.0(60.4)**	***24.6(18.9)***	**34.3(22.1)**
	(5) APacc/MLvel	RMSE Abs Bias	***39.3***	**45.2**	75.6	74.4	46.5
			***35.6(16.6)***	**43.3(13.2)**	64.5(39.4)	69.2(27.2)	44.6(13.2)

**Table 4:** Stride time RMSE and mean absolute bias values (standard deviation) for the straight over ground walking task and the obstacle navigation task reported in milliseconds. Algorithm type is represented by each row; sensor location is represented in each column. Bold text indicates an RMSE/mean absolute bias value was lowest across algorithms for a given sensor location. Italicized text indicates an RMSE/mean absolute bias value was lowest across sensor locations for a given algorithm.

		Straight over Ground Walking					
Stride Time	Algorithm Type		Dorsal Foot	Heel	Lower Lateral Shank	Middle Lateral Shank	Shin Bone
	(1) Velocity Minima	RMSE Abs Bias	17.2	***9.2***	28.3	12.1	15.7
			11.5(12.8)	***7.2(5.6)***	18.6(21.4)	9.4(7.6)	10.7(11.5)
	(2) Velocity Zero Crossing	RMSE Abs Bias	***12.3***	12.4	12.6	12.8	**12.8**
			***9.7(7.5)***	9.9(7.5)	10.1(7.6)	10.2(7.7)	**10.2(7.6)**
	(3) SIacc/APacc	RMSE Abs Bias	35.0	33.6	45.5	53.3	*28.0*
			21.8(27.4)	23.3(24.2)	29.6(34.6)	40.6(34.6)	*21.4(18.1)*
	(4) MLvel/APacc	RMSE Abs Bias	14.8	13.4	**11.7**	***10.7***	34.6
			10.5(10.5)	9.8(9.1)	**9.4(6.9)**	***8.8(6.1)***	24.9(24.1)
	(5) APacc/MLvel	RMSE Abs Bias	29.1	*20.1*	70.4	52.9	52.3
			18.6(22.4)	*11.0(16.8)*	51.9(47.5)	33.3(41.0)	33.7(40.0)
		**Obstacle navigation**					
	Algorithm Type		Dorsal Foot	Heel	Lower Lateral Shank	Middle Lateral Shank	Shin Bone
	(1) Velocity Minima	RMSE Abs Bias	19.5	***9.4***	33.3	15.5	21.6
			11.9(15.4)	***5.6(7.5)***	22.0(25.0)	10.5(11.4)	13.1(17.2)
	(2) Velocity Zero Crossing	RMSE Abs Bias	**15**	*14.3*	15	14.8	**15.2**
			**11.2(10)**	*10.4(9.8)*	11.3(9.9)	11.1(9.8)	**11.4(10.1)**
	(3) SIacc/APacc	RMSE Abs Bias	33.6	44.0	43.8	60.5	*32.6*
			22.1(25.4)	34.3(27.5)	30.9(31)	44.1(41.5)	*20.3(25.6)*
	(4) MLvel/APacc	RMSE Abs Bias	18.0	*10.3*	**13.0**	**13.2**	41.9
			11.6(13.8)	*6.8(7.8)*	**9.6(8.7)**	**9.4(9.3)**	29.7(29.6)
	(5) APacc/MLvel	RMSE Abs Bias	24.7	*18.7*	77.2	56.2	47.2
			17.2(17.7)	*8.8(16.5)*	58.5(50.3)	37.2(42.1)	31.1(35.5)

## Data Availability

The data used for the current analysis are available online at: https://github.com/dbp-osel/IMU-Sensor-Placement-Optimization.
